# Supplementary material for: Reversibility of quantum resources through probabilistic protocols
Source: Nat Commun. 2024 Apr 17;15:3096. doi: 10.1038/s41467-024-47243-2 (PMC11024169; doi:10.1038/s41467-024-47243-2)
Supplement: Supplementary file 1 — Supplementary Information [file 41467_2024_47243_MOESM1_ESM.pdf]

Reversibility of quantum resources through probabilistic protocols
— Supplementary information —

Bartosz Regula* and Ludovico Lami†

CONTENTS

I. Notation and basic properties 1

II. Probabilistic reversibility of general quantum resources 5

III. Equivalence of strong converse and probabilistic distillation in entanglement theory 9

IV. Irreversibility of entanglement theory under probabilistic non-entangling operations 11

References 17

I. NOTATION AND BASIC PROPERTIES

We begin by clarifying the assumptions and definitions used in this work.

A. Free operations and free states

Throughout this paper paper we assume that the underlying Hilbert space has finite dimension. When discussing many-copy state transformations, we assume that each n-copy space of quantum states has its own associated set of ***free states*** $\mathbb{F}_n$. We often use $\mathbb{F}$ to refer to the whole family $(\mathbb{F}_n)_n$ for simplicity.

Following [1, 2], we will assume the following basic axioms about the resource theory in consideration:

Axiom I. Each $\mathbb{F}_n$ is convex and closed.

Axiom II. There exists a full-rank state σ such that $\sigma^{\otimes n} \in \mathbb{F}_n$ for all n.

Axiom III. The sets $\mathbb{F}_n$ are closed under partial trace: if $\sigma \in \mathbb{F}_{n+1}$, then $\operatorname{Tr}_k \sigma \in \mathbb{F}_n$ for every $k \in \{1, \ldots, n+1\}$.

Axiom IV. The sets $\mathbb{F}_n$ are closed under tensor product: if $\sigma \in \mathbb{F}_n$ and $\sigma' \in \mathbb{F}_m$, then $\sigma \otimes \sigma' \in \mathbb{F}_{n+m}$.

The motivation for these assumptions is so that the theory is well behaved asymptotically [1–3].[1] These undemanding conditions are obeyed by the vast majority of practically relevant resource theories. In addition, we will need one more assumption that was implicitly used in [1] and later appeared explicitly in [2]:

Axiom V. The regularised relative entropy $D^{\infty}_{\mathbb{F}}(\rho) = \lim_{n\to\infty} \frac{1}{n} \min_{\sigma\in\mathbb{F}_n} D(\rho^{\otimes n}\|\sigma)$ is non-zero for all states $\rho \notin \mathbb{F}$.

Once again, Axiom V holds true for most resources encountered in practice, in particular for quantum entanglement [4]. However, there do exist theories in which $D^{\infty}_{\mathbb{F}}$ vanishes for some (or even all) states $\rho \notin \mathbb{F}$, e.g. the theory of asymmetry [5]. The standard reversibility conjectures do not apply to such theories [2].

We use CPTP to denote completely positive and trace-preserving maps (quantum channels), and CPTNI to denote completely positive and trace–non-increasing maps (probabilistic quantum operations). A ***probabilistic protocol*** is a collection of CPTNI maps $\{\mathcal{E}^{(i)}\}_i$ that forms a valid

* bartosz.regula@gmail.com
† ludovico.lami@gmail.com

[1] In [1, 2], an additional axiom of permutation invariance was imposed. We do not need this here.

quantum instrument, i.e. the overall transformation $\sum_i \mathcal{E}^{(i)}$ is trace preserving. The latter condition simply means that the outcome probabilities necessarily add up to one. Given some class of ***free operations*** $\mathbb{O}$, we say that a protocol is free if $\mathcal{E}^{(i)} \in \mathbb{O}\ \forall i$. When discussing transformations between two fixed states $\rho \to \omega$, we may coarse-grain the outcomes of the instrument and consider only two-outcome protocols $\{\mathcal{E}^{(i)}\}_{i\in\{0,1\}}$ — either the protocol is successful in performing the given transformation, or it fails. We can then say that ρ can be converted to ω probabilistically if there exists a free protocol $\{\mathcal{E}^{(i)}\}_{i\in\{0,1\}}$ such that $\mathcal{E}^{(0)}(\rho) \propto \omega$.

Resource non-generating (RNG) operations are the maps which can never transform a resourceless state into a resourceful one:

$$\mathbb{O}_{\mathrm{RNG}} := \left\{ \mathcal{E} \in \mathrm{CPTNI} \;\middle|\; \frac{\mathcal{E}(\sigma)}{\operatorname{Tr}\mathcal{E}(\sigma)} \in \mathbb{F}\ \forall \sigma \in \mathbb{F} \right\}. \tag{S1}$$

Their approximate variant is defined as

$$\mathbb{O}_{\mathrm{RNG},\delta} := \left\{ \mathcal{E} \in \mathrm{CPTNI} \;\middle|\; R^g_{\mathbb{F}}\left(\frac{\mathcal{E}(\sigma)}{\operatorname{Tr}\mathcal{E}(\sigma)}\right) \leq \delta\ \forall \sigma \in \mathbb{F} \right\}. \tag{S2}$$

We note here that the latter definition depends on the choice of the measure with which one quantifies the generated resources — in this case, this is the ***generalised robustness*** [6]

$$\begin{aligned} R^g_{\mathbb{F}}(\rho) &:= \inf\left\{ \lambda \in \mathbb{R}_+ \;\middle|\; \frac{\rho + \lambda\omega}{1+\lambda} \in \mathbb{F},\ \omega \in \mathbb{D} \right\} \\ &= \inf\left\{ \lambda \in \mathbb{R}_+ \;\middle|\; \rho \leq (\lambda+1)\sigma,\ \sigma \in \mathbb{F} \right\}. \end{aligned} \tag{S3}$$

This quantity can also be identified with the max-relative entropy of a resource [3], $D_{\max,\mathbb{F}}(\rho) := \log\left(1 + R^g_{\mathbb{F}}(\rho)\right)$. Other choices of measures may lead to completely different asymptotic behaviour [7], and our choice here is motivated by the framework of [8, 9] that conjectured reversibility of quantum resources under operations defined in this way.

Under resource non-generating transformations $\mathbb{O}_{\mathrm{RNG}}$ (or $\mathbb{O}_{\mathrm{RNG},\delta}$), the existence of a probabilistic protocol that performs a transformation $\rho \to \omega$ is fully equivalent to the existence of a map $\mathcal{E} \in \mathbb{O}_{\mathrm{RNG}}$ such that $\mathcal{E}(\rho)/\operatorname{Tr}\mathcal{E}(\rho) = \omega$. This is because any such map can always be completed to a free instrument: we simply define $\mathcal{E}'(X) := [\operatorname{Tr} X - \operatorname{Tr}\mathcal{E}(X)]\,\sigma$ for some $\sigma \in \mathbb{F}$, yielding $(\mathcal{E}, \mathcal{E}') \in \mathbb{O}$. For other sets of operations, it may be the case that a map $\mathcal{E} \in \mathbb{O}$ cannot always be completed to a free instrument $(\mathcal{E}, \mathcal{E}')$ — e.g. for separable operations in entanglement theory [10] or stabiliser operations in the theory of magic states [11] — so more care may need to be taken when discussing probabilistic transformations.

B. Transformation rates

Given a class of completely positive and trace non-increasing maps $\mathbb{O}$, the various transformation rates that we study are defined as follows.

The ***deterministic conversion rate*** is

$$r_{p=1}(\rho \xrightarrow[\mathbb{O}]{} \omega) := \sup_{(\mathcal{E}_n)_n} \left\{ r \;\middle|\; \lim_{n\to\infty} F\left(\mathcal{E}_n(\rho^{\otimes n}),\ \omega^{\otimes \lfloor rn \rfloor}\right) = 1, \quad \mathcal{E}_n \in \mathbb{O} \cap \mathrm{CPTP} \right\}, \tag{S4}$$

where the optimisation is over all sequences $(\mathcal{E}_n)_n$ of maps satisfying the given constraints. Here, $F(\rho,\sigma) := \left\|\sqrt{\rho}\sqrt{\sigma}\right\|_1^2$ is the fidelity.

The ***probabilistic rate with non-vanishing probability of success*** is

$$\begin{aligned} r_{p>0}(\rho \xrightarrow[\mathbb{O}]{} \omega) := \sup_{(\mathcal{E}_n)_n} \Bigg\{ r \;\Bigg|\; & \lim_{n\to\infty} F\left(\frac{\mathcal{E}_n(\rho^{\otimes n})}{\operatorname{Tr}\mathcal{E}_n(\rho^{\otimes n})},\ \omega^{\otimes \lfloor rn \rfloor}\right) = 1, \\ & \mathcal{E}_n \in \mathbb{O}, \quad \liminf_{n\to\infty} \operatorname{Tr}\mathcal{E}_n(\rho^{\otimes n}) > 0 \Bigg\}. \end{aligned} \tag{S5}$$

We refer to $1 - F\left(\frac{\mathcal{E}_n(\rho^{\otimes n})}{\operatorname{Tr}\mathcal{E}_n(\rho^{\otimes n})},\ \omega^{\otimes \lfloor rn \rfloor}\right)$ as the ***transformation error*** and to $\operatorname{Tr}\mathcal{E}_n(\rho^{\otimes n})$ as the ***transformation probability***.

	Standard	Strong converse †	Stronger strong converse ‡
Deterministic	$r_{p=1}(\rho \xrightarrow[\mathbb{O}]{} \omega)$ (S4)	$r^{\dagger}_{p=1}(\rho \xrightarrow[\mathbb{O}]{} \omega)$ (S6)	$r^{\ddagger}_{p=1}(\rho \xrightarrow[\mathbb{O}]{} \omega)$ (S7)
Probabilistic	$r_{p>0}(\rho \xrightarrow[\mathbb{O}]{} \omega)$ (S5)	$r^{\dagger}_{p>0}(\rho \xrightarrow[\mathbb{O}]{} \omega)$ (S8)	$r^{\ddagger}_{p>0}(\rho \xrightarrow[\mathbb{O}]{} \omega)$ (S9)

TABLE I: The transformation rates defined by Eqs. (S4)–(S9).

The ***deterministic strong converse rate*** is

$$\begin{aligned} r^{\dagger}_{p=1}(\rho \xrightarrow[\mathbb{O}]{} \omega) &:= \sup_{(\mathcal{E}_n)_n} \left\{ r \;\middle|\; \liminf_{n\to\infty} F\Big(\mathcal{E}_n(\rho^{\otimes n}),\, \omega^{\otimes \lfloor rn \rfloor}\Big) > 0, \quad \mathcal{E}_n \in \mathbb{O} \cap \mathrm{CPTP} \right\}. \\ &= \inf \left\{ r' \;\middle|\; \liminf_{n\to\infty} \sup_{\mathcal{E}_n \in \mathbb{O}\cap \mathrm{CPTP}} F\Big(\mathcal{E}_n(\rho^{\otimes n}),\, \omega^{\otimes \lfloor r'n \rfloor}\Big) = 0 \right\}. \end{aligned} \tag{S6}$$

We note here that there are differences in the precise definition of a strong converse in the literature, and stronger definitions can sometimes be encountered. One such variant is

$$\begin{aligned} r^{\ddagger}_{p=1}(\rho \xrightarrow[\mathbb{O}]{} \omega) &:= \sup_{(\mathcal{E}_n)_n} \left\{ r \;\middle|\; \limsup_{n\to\infty} F\Big(\mathcal{E}_n(\rho^{\otimes n}),\, \omega^{\otimes \lfloor rn \rfloor}\Big) > 0, \quad \mathcal{E}_n \in \mathbb{O} \cap \mathrm{CPTP} \right\} \\ &= \inf \left\{ r' \;\middle|\; \lim_{n\to\infty} \sup_{\mathcal{E}_n \in \mathbb{O}\cap \mathrm{CPTP}} F\Big(\mathcal{E}_n(\rho^{\otimes n}),\, \omega^{\otimes \lfloor r'n \rfloor}\Big) = 0 \right\}. \end{aligned} \tag{S7}$$

The difference between $r^{\dagger}$ and $r^{\ddagger}$ is that the former maximises over rates which are truly achievable, albeit with a large error; in other words, for all sufficiently large n, the fidelity with the target state must be larger than some non-zero constant. On the other hand, the maximisation in (S7) only requires that the fidelity be non-zero infinitely often. It can be noticed that $r^{\dagger}_{p=1} \leq r^{\ddagger}_{p=1}$ in general, although we do not know if this inequality can ever be strict when considering i.i.d. state transformations as we do here. We choose to employ the definition of $r^{\dagger}$ as in Eq. (S6) as it will lead to tighter statements of our results. On the other hand, we will sometimes use $r^{\ddagger}$ to provide potentially stronger upper (converse) bounds on rates. A reader not interested in these technicalities may safely ignore the difference between the two definitions.

For completeness, we will also briefly consider the ***strong converse probabilistic rate***

$$\begin{aligned} r^{\dagger}_{p>0}(\rho \xrightarrow[\mathbb{O}]{} \omega) := \sup_{(\mathcal{E}_n)_n} \Bigg\{ r \;\Bigg|\; &\liminf_{n\to\infty} F\left(\frac{\mathcal{E}_n(\rho^{\otimes n})}{\operatorname{Tr}\mathcal{E}_n(\rho^{\otimes n})},\, \omega^{\otimes \lfloor rn \rfloor}\right) > 0, \\ &\mathcal{E}_n \in \mathbb{O}, \quad \liminf_{n\to\infty} \operatorname{Tr}\mathcal{E}_n(\rho^{\otimes n}) > 0 \Bigg\}, \end{aligned} \tag{S8}$$

together with its variant

$$\begin{aligned} r^{\ddagger}_{p>0}(\rho \xrightarrow[\mathbb{O}]{} \omega) := \sup_{(\mathcal{E}_n)_n} \Bigg\{ r \;\Bigg|\; &\limsup_{n\to\infty} F\left(\frac{\mathcal{E}_n(\rho^{\otimes n})}{\operatorname{Tr}\mathcal{E}_n(\rho^{\otimes n})},\, \omega^{\otimes \lfloor rn \rfloor}\right) > 0, \\ &\mathcal{E}_n \in \mathbb{O}, \quad \liminf_{n\to\infty} \operatorname{Tr}\mathcal{E}_n(\rho^{\otimes n}) > 0 \Bigg\}, \end{aligned} \tag{S9}$$

where the only difference between Eq. (S8) and Eq. (S9), just like for Eq. (S6) and Eq. (S7), is the presence of a lim inf or a lim sup over the achievable transformation fidelities.

Finally, our main object of study are probabilistic transformation rates under ***asymptotically resource–non-generating transformations*** $\mathbb{O}_{\mathrm{ARNG}}$, which are sequences of operations in $\mathbb{O}_{\mathrm{RNG},\delta_n}$ such that $\delta_n \xrightarrow{n\to\infty} 0$. Specifically,

$$\begin{aligned} r_{p>0}(\rho \xrightarrow[\mathrm{ARNG}]{} \omega) := \sup_{(\mathcal{E}_n)_n} \Bigg\{ r \;\Bigg|\; &\lim_{n\to\infty} F\left(\frac{\mathcal{E}_n(\rho^{\otimes n})}{\operatorname{Tr}\mathcal{E}_n(\rho^{\otimes n})},\, \omega^{\otimes \lfloor rn \rfloor}\right) = 1, \\ &\lim_{n\to\infty} \sup_{\sigma\in\mathbb{F}_n} R^g_{\mathbb{F}_{\lfloor rn \rfloor}}\left(\frac{\mathcal{E}_n(\sigma)}{\operatorname{Tr}\mathcal{E}_n(\sigma)}\right) = 0, \quad \liminf_{n\to\infty} \operatorname{Tr}\mathcal{E}_n(\rho^{\otimes n}) > 0 \Bigg\}. \end{aligned} \tag{S10}$$

When employing the above definitions, we will make use of the fact that, due to the Fuchs–van de Graaf inequalities [12], the transformation error can be equivalently defined with the trace distance $\frac{1}{2}\left\|\rho-\sigma\right\|_1$:

$$\begin{aligned}
\lim_{n\to\infty} F\left(\frac{\mathcal{E}_n(\rho^{\otimes n})}{\operatorname{Tr}\mathcal{E}_n(\rho^{\otimes n})},\, \omega^{\otimes\lfloor rn\rfloor}\right) = 1 \quad&\Longleftrightarrow\quad \lim_{n\to\infty} \frac{1}{2}\left\|\frac{\mathcal{E}_n(\rho^{\otimes n})}{\operatorname{Tr}\mathcal{E}_n(\rho^{\otimes n})} - \omega^{\otimes\lfloor rn\rfloor}\right\|_1 = 0,\\
\liminf_{n\to\infty} F\left(\frac{\mathcal{E}_n(\rho^{\otimes n})}{\operatorname{Tr}\mathcal{E}_n(\rho^{\otimes n})},\, \omega^{\otimes\lfloor rn\rfloor}\right) > 0 \quad&\Longleftrightarrow\quad \limsup_{n\to\infty} \frac{1}{2}\left\|\frac{\mathcal{E}_n(\rho^{\otimes n})}{\operatorname{Tr}\mathcal{E}_n(\rho^{\otimes n})} - \omega^{\otimes\lfloor rn\rfloor}\right\|_1 < 1.
\end{aligned} \tag{S11}$$

The relations between the different transformation rates are encapsulated in the following lemma.

Lemma S1. *Let $\mathbb{O}$ be any class of free operations such that, for an instrument $\{\mathcal{E}^{(i)}\}_i$ with $\mathcal{E}^{(i)} \in \mathbb{O}$, it holds that $\sum_i \mathcal{E}^{(i)} \in \mathbb{O}$. This can be, for instance, the classes $\mathbb{O}_{\mathrm{RNG}}$ or $\mathbb{O}_{\mathrm{RNG},\delta}$ in any convex resource theory, or any suitable class of free operations in entanglement theory such as LOCC.*
Then, for all quantum states ρ and ω, the transformation rates satisfy

$$\begin{aligned}
r_{p=1}(\rho \underset{\mathbb{O}}{\to} \omega) \;\le\; r_{p>0}(\rho \underset{\mathbb{O}}{\to} \omega) \;\le\; r^{\dagger}_{p=1}(\rho \underset{\mathbb{O}}{\to} \omega) \;&=\; r^{\dagger}_{p>0}(\rho \underset{\mathbb{O}}{\to} \omega)\\
\le\; r^{\ddagger}_{p=1}(\rho \underset{\mathbb{O}}{\to} \omega) \;&=\; r^{\ddagger}_{p>0}(\rho \underset{\mathbb{O}}{\to} \omega).
\end{aligned} \tag{S12}$$

Remark. Let us comment briefly on the assumption of this lemma. If there exists a classical system C with an orthonormal basis $\{|i\rangle_C\}_i$ such that each element of the basis is a free state, one can equivalently understand the realisation of any probabilistic protocol $\{\mathcal{E}^{(i)}\}_i$ as a CPTP map

$$\rho \mapsto \sum_i \mathcal{E}^{(i)}(\rho) \otimes |i\rangle\langle i|_C \tag{S13}$$

where the classical system is used to keep track of the probabilistic outcome of the protocol. If we are free to 'forget' this outcome — that is, if tracing out the classical system is an allowed operation — then the given set $\mathbb{O}$ satisfies the conditions of the lemma. This is a very weak assumption that is satisfied by essentially all types of free operations in the majority of resource theories, but there are special cases in which it may not be obeyed, e.g. for thermal operations in the theory of thermodynamics [13].

Proof. As deterministic protocols are a special case of probabilistic ones, the first inequality is immediate. For the second inequality, assume that there exists a sequence of free probabilistic operations $(\mathcal{E}_n)_n$ which performs the transformation $\rho \to \omega$ at a rate r with error ε_n such that $\lim_{n\to\infty} \varepsilon_n = 0$ and probability p_n such that $\liminf_{n\to\infty} p_n = p > 0$. By definition of a probabilistic protocol, there exist operations $\mathcal{E}'_n \in \mathbb{O}$ which complete each $\mathcal{E}_n$ to a valid quantum instrument, that is, such that

$$\mathcal{D}_n(X) \coloneqq \mathcal{E}_n(X) + \mathcal{E}'_n(X) \tag{S14}$$

is trace preserving. (When $\mathbb{O}$ denotes resource non-generating operations, we can simply take $\mathcal{E}'_n(X) = [\operatorname{Tr} X - \operatorname{Tr}\mathcal{E}_n(X)]\,\sigma_n$ for some $\sigma_n \in \mathbb{F}$.) By hypothesis, we have that $\mathcal{D}_n \in \mathbb{O}$.
Since $p_n = \operatorname{Tr}\mathcal{E}_n(\rho^{\otimes n})$, the concavity of the fidelity then gives

$$\begin{aligned}
\liminf_{n\to\infty} F\Big(\mathcal{D}_n(\rho^{\otimes n}), \omega^{\otimes\lfloor rn\rfloor}\Big) &\ge \liminf_{n\to\infty}\, p_n\, F\left(\frac{\mathcal{E}_n(\rho^{\otimes n})}{p_n},\, \omega^{\otimes\lfloor rn\rfloor}\right)\\
&= \liminf_{n\to\infty}\, p_n(1-\varepsilon_n)\\
&= p > 0,
\end{aligned} \tag{S15}$$

hence $r \le r^{\dagger}_{p=1}(\rho \to \omega)$.

Let now r be any feasible rate for $r^{\dagger}_{p>0}(\rho \underset{\mathbb{O}}{\to} \omega)$, that is, such that there exists a sequence of free probabilistic operations $(\mathcal{E}_n)_n$ with non-vanishing error $\limsup_{n\to\infty} \varepsilon_n = \varepsilon < 1$ and probability of success $\liminf_{n\to\infty} p_n = p > 0$. The same argument as above can be used to construct a deterministic protocol with

$$\liminf_{n\to\infty} F\Big(\mathcal{D}_n(\rho^{\otimes n}), \omega^{\otimes\lfloor rn\rfloor}\Big) \ge \liminf_{n\to\infty}\, p_n(1-\varepsilon_n) \ge p(1-\varepsilon) > 0, \tag{S16}$$

yielding $r \leq r^{\dagger}_{p=1}(\rho \xrightarrow[\mathbb{O}]{} \omega)$ and hence $r^{\dagger}_{p>0} \leq r^{\dagger}_{p=1}$. Since the opposite inequality between the strong converse rates follows straightforwardly from the fact that any deterministic protocol is a special case of a probabilistic one, the two rates must be equal.

The inequality $r^{\dagger}_{p>0} \leq r^{\ddagger}_{p>0}$ follows immediately by comparing (S8) and (S9). The equivalence between the stronger strong converse rates $r^{\ddagger}_{p>0}$ and $r^{\ddagger}_{p=1}$ proceeds analogously, where we now use that

$$\limsup_{n\to\infty} F\Big(\mathcal{D}_n(\rho^{\otimes n}), \omega^{\otimes \lfloor rn \rfloor}\Big) \geq \liminf_{n\to\infty} p_n \limsup_{n\to\infty} (1-\varepsilon_n) \geq p(1-\varepsilon) > 0. \tag{S17}$$

This completes the proof. ∎

II. PROBABILISTIC REVERSIBILITY OF GENERAL QUANTUM RESOURCES

Theorem 1. *In any resource theory satisfying axioms I–V of Sec. I A, it holds that*

$$r_{p>0}(\rho \xrightarrow[\text{ARNG}]{} \omega) = \frac{D^{\infty}_{\mathbb{F}}(\rho)}{D^{\infty}_{\mathbb{F}}(\omega)}. \tag{S18}$$

Proof. *Converse.* — We will often use the logarithmic variant of $R^{g}_{\mathbb{F}}$, for which we recall the standard notation

$$D_{\max,\mathbb{F}}(\rho) = \log\Big(1 + R^{g}_{\mathbb{F}}(\rho)\Big). \tag{S19}$$

Assume that r is an achievable probabilistic rate, that is, there exists a sequence of protocols $(\mathcal{E}_n)_n$ with $\mathcal{E}_n \in \mathbb{O}_{\text{RNG}_{\delta_n}}$ such that $\frac12 \left\| \frac{\mathcal{E}_n(\rho^{\otimes n})}{\operatorname{Tr}\mathcal{E}_n(\rho^{\otimes n})} - \omega^{\otimes \lfloor rn \rfloor} \right\|_1 =: \varepsilon_n$ with $\lim_{n\to\infty} \varepsilon_n = 0$, $\liminf_{n\to\infty} \operatorname{Tr}\mathcal{E}_n(\rho^{\otimes n}) =: p > 0$, and furthermore $\lim_{n\to\infty} \delta_n = 0$. For all $\zeta < p$ and all sufficiently large n, it then holds that $\zeta/\operatorname{Tr}\mathcal{E}_n(\rho^{\otimes n}) + \varepsilon_n < 1$. Letting

$$D^{\zeta}_{\max,\mathbb{F}}(\rho) := \min_{\frac12\|\rho-\rho'\|_1 \leq \zeta} D_{\max,\mathbb{F}}(\rho'), \tag{S20}$$

we use the probabilistic monotonicity of the max-relative entropy (Lemma S3 below) to get

$$\begin{aligned} D^{\zeta}_{\max,\mathbb{F}}(\rho^{\otimes n}) &\geq D^{\zeta/\operatorname{Tr}\mathcal{E}_n(\rho^{\otimes n})}_{\max,\mathbb{F}}\left(\frac{\mathcal{E}_n(\rho^{\otimes n})}{\operatorname{Tr}\mathcal{E}_n(\rho^{\otimes n})}\right) + \log\left(\operatorname{Tr}\mathcal{E}_n(\rho^{\otimes n}) - \zeta\right) - \log(1+\delta_n) \\ &\geq D^{\zeta/\operatorname{Tr}\mathcal{E}_n(\rho^{\otimes n})+\varepsilon_n}_{\max,\mathbb{F}}\left(\omega^{\otimes\lfloor rn \rfloor}\right) + \log\left(\operatorname{Tr}\mathcal{E}_n(\rho^{\otimes n}) - \zeta\right) - \log(1+\delta_n). \end{aligned} \tag{S21}$$

Here, in the last line we used the fact that $\frac12 \left\|\tau - \omega^{\otimes\lfloor rn\rfloor}\right\|_1 \leq \frac12 \left\|\tau - \frac{\mathcal{E}_n(\rho^{\otimes n})}{\operatorname{Tr}\mathcal{E}_n(\rho^{\otimes n})}\right\|_1 + \frac12 \left\|\frac{\mathcal{E}_n(\rho^{\otimes n})}{\operatorname{Tr}\mathcal{E}_n(\rho^{\otimes n})} - \omega^{\otimes\lfloor rn\rfloor}\right\|_1$ for any state τ.

Applying the above, we obtain that

$$\begin{aligned} \inf_{(\zeta_n)_n}\left\{ \limsup_{n\to\infty} \frac1n D^{\zeta_n}_{\max,\mathbb{F}}(\rho^{\otimes n}) \,\middle|\, \lim_{n\to\infty} \zeta_n = 0 \right\} &\geq \inf_{(\zeta_n)_n}\left\{ \limsup_{n\to\infty} \frac1n D^{\zeta_n/\operatorname{Tr}\mathcal{E}_n(\rho^{\otimes n})+\varepsilon_n}_{\max,\mathbb{F}}\left(\omega^{\otimes\lfloor rn\rfloor}\right) \,\middle|\, \lim_{n\to\infty} \zeta_n = 0 \right\} \\ &\geq \inf_{(\eta_n)_n}\left\{ \limsup_{n\to\infty} \frac1n D^{\eta_n}_{\max,\mathbb{F}}\left(\omega^{\otimes\lfloor rn\rfloor}\right) \,\middle|\, \lim_{n\to\infty} \eta_n = 0 \right\}, \end{aligned} \tag{S22}$$

where in the third line we observed that $\zeta_n/\operatorname{Tr}\mathcal{E}_n(\rho^{\otimes n}) + \varepsilon_n$ tends to 0 for any sequence $(\zeta_n)_n$ tending to 0. Using the asymptotic equipartition property [1, 3]

$$\inf_{(\zeta_n)_n}\left\{ \limsup_{n\to\infty} \frac1n D^{\zeta_n}_{\max,\mathbb{F}}(\omega^{\otimes\lfloor rn\rfloor}) \,\middle|\, \lim_{n\to\infty} \zeta_n = 0 \right\} = r\, D^{\infty}_{\mathbb{F}}(\omega) \tag{S23}$$

on both sides of (S22) concludes the converse part of the Theorem.

Achievability. — From the results of Brandão–Plenio [1], and in particular from the aforementioned equipartition property, we know two things:

1) ([1, Proposition II.1 and Corollary III.2]; also [3, Theorem 1]) For any ω, any $r > 0$ and any $c > D_{\mathbb{F}}^{\infty}(\omega)$, there exists a sequence of states $(\omega_n)_n$ such that $F\big(\omega_n, \omega^{\otimes \lfloor rn \rfloor}\big) \geq 1 - \varepsilon_n$ with $\lim_{n\to\infty} \varepsilon_n = 0$ and such that

$$\limsup_{n\to\infty} \frac{1}{n} \log\left(1 + R_{\mathbb{F}}^{g}(\omega_n)\right) = \limsup_{n\to\infty} \frac{1}{n} D_{\max,\mathbb{F}}(\omega_n) = cr. \tag{S24}$$

2) ([1, Corollary III.3; cf. also proof of Theorem I therein]) For any ρ and any $d < D_{\mathbb{F}}^{\infty}(\rho)$, there exists a sequence of POVM elements $(A_n)_n$ satisfying $0 \leq A_n \leq \mathbb{1}$ for all n, and moreover $\operatorname{Tr}(A_n \rho^{\otimes n}) \geq 1 - \delta_n$, where the sequence $(\delta_n)_n$ is such that

$$\limsup_{n\to\infty} \delta_n = \delta < 1 \tag{S25}$$

and[2]

$$\liminf_{n\to\infty} -\frac{1}{n} \log \sup_{\sigma\in\mathbb{F}_n} \operatorname{Tr}(A_n\sigma) = d. \tag{S26}$$

We define the shorthands

$$\begin{aligned} a_n &:= \sup_{\sigma\in\mathbb{F}_n} \operatorname{Tr}(A_n\sigma), \\ \lambda_n &:= 1 + R_{\mathbb{F}}^{g}(\omega_n). \end{aligned} \tag{S27}$$

Let us use the above to construct a probabilistic protocol which transforms n copies of ρ to $\lfloor rn \rfloor$ copies of ω for some r. We start by defining the sequence of maps

$$\mathcal{E}_n(X) := \operatorname{Tr}(A_n X)\, \omega_n + \mu_n \operatorname{Tr}[(\mathbb{1} - A_n)X]\, \pi_n, \tag{S28}$$

where $\mu_n \in [0,1]$ are some parameters to be chosen later, ω_n are the states appearing in (S24), and π_n are states such that

$$\frac{\omega_n + (\lambda_n - 1)\pi_n}{\lambda_n} = \sigma_n \in \mathbb{F}_{\lfloor rn \rfloor}, \tag{S29}$$

which exist by definition of $R_{\mathbb{F}}^{g}$ and closedness of $\mathbb{F}$. The maps are clearly completely positive and trace non-increasing by construction.

We will now show that, for any choice of a sequence $(\mu_n)_n$ such that

$$\mu_n \geq \frac{\lambda_n - 1}{a_n^{-1} - 1} \tag{S30}$$

for all n, the sequence of maps $(\mathcal{E}_n)_n$ is asymptotically resource non-generating. This can be seen by observing that, for any $\sigma \in \mathbb{F}_n$, it holds that

$$\begin{aligned} \mathcal{E}_n(\sigma) &= \operatorname{Tr}(A_n\sigma)\, \omega_n + \mu_n (1 - \operatorname{Tr}(A_n\sigma))\pi_n \\ &= \operatorname{Tr}(A_n\sigma)\, \lambda_n \frac{\omega_n + (\lambda_n - 1)\pi_n}{\lambda_n} + \left[\mu_n\big(1 - \operatorname{Tr}(A_n\sigma)\big) - \operatorname{Tr}(A_n\sigma)\,(\lambda_n - 1)\right]\pi_n \\ &\overset{\text{(i)}}{\leq} \operatorname{Tr}(A_n\sigma)\, \lambda_n\, \sigma_n + \left[\mu_n\big(1 - \operatorname{Tr}(A_n\sigma)\big) - \operatorname{Tr}(A_n\sigma)\,(\lambda_n - 1)\right] \frac{\lambda_n}{\lambda_n - 1}\sigma_n \\ &\overset{\text{(ii)}}{\leq} \operatorname{Tr}(A_n\sigma)\, \lambda_n \frac{\lambda_n}{\lambda_n - 1}\, \sigma_n + \left[\mu_n\big(1 - \operatorname{Tr}(A_n\sigma)\big) - \operatorname{Tr}(A_n\sigma)\,(\lambda_n - 1)\right] \frac{\lambda_n}{\lambda_n - 1}\sigma_n \\ &= \left[\mu_n\big(1 - \operatorname{Tr}(A_n\sigma)\big) + \operatorname{Tr}(A_n\sigma)\right] \frac{\lambda_n}{\lambda_n - 1}\sigma_n \\ &= \left[\operatorname{Tr}\mathcal{E}_n(\sigma)\right] \frac{\lambda_n}{\lambda_n - 1}\sigma_n. \end{aligned} \tag{S31}$$

---

[2] The proof of [1] actually makes the stronger statement that we can take $-\frac{1}{n}\log \sup_{\sigma\in\mathbb{F}_n} \operatorname{Tr}(A_n\sigma) = d$ for all n. We do not need this here, hence we leave the statement in the above more general form.

Here, in (i) we used the fact that $\pi_n \le \frac{\lambda_n}{\lambda_n - 1}\sigma_n$, which follows from Eq. (S29), as well as that $\mu_n\big(1 - \operatorname{Tr}(A_n\sigma)\big) - \operatorname{Tr}(A_n\sigma)\,(\lambda_n - 1) \ge 0$, which is due to the assumption in Eq. (S30). In (ii), we simply used that $\frac{\lambda_n}{\lambda_n - 1} \ge 1$. The above guarantees that

$$\lim_{n\to\infty} R^g_{\mathbb{F}}\left(\frac{\mathcal{E}_n(\sigma)}{\operatorname{Tr}\mathcal{E}_n(\sigma)}\right) \le \lim_{n\to\infty}\left(\frac{\lambda_n}{\lambda_n - 1} - 1\right) = 0, \tag{S32}$$

where the last identity follows from the observation that λ_n diverges exponentially, as is apparent from (S24) and (S27).

Crucially, the probability of success of these operations satisfies

$$\begin{aligned} p_n = \operatorname{Tr}\mathcal{E}_n(\rho^{\otimes n}) &= \operatorname{Tr}\left(A_n\rho^{\otimes n}\right) + \mu_n\left(1 - \operatorname{Tr}\left(A_n\rho^{\otimes n}\right)\right) \\ &\ge 1 - \delta_n(1 - \mu_n), \end{aligned} \tag{S33}$$

while the transformation fidelity is at least

$$\begin{aligned} F\left(\frac{\mathcal{E}_n(\rho^{\otimes n})}{\operatorname{Tr}\mathcal{E}_n(\rho^{\otimes n})}, \omega^{\otimes\lfloor rn\rfloor}\right) &\ge \frac{\operatorname{Tr}\left(A_n\rho^{\otimes n}\right)}{\operatorname{Tr}\mathcal{E}_n(\rho^{\otimes n})} F(\omega_n, \omega^{\otimes\lfloor rn\rfloor}) \\ &\ge \frac{1 - \delta_n}{1 - \delta_n + \mu_n\delta_n}(1 - \varepsilon_n), \end{aligned} \tag{S34}$$

where the first line follows by the concavity of the fidelity. It follows that, by decreasing μ_n, we can decrease the error in this transformation; on the other hand, the probability of success will never go below $1 - \delta > 0$ no matter how much μ_n is decreased, since $\delta_n \xrightarrow{n\to\infty} \delta < 1$.

Let us then fix

$$\mu_n := \frac{\lambda_n - 1}{a_n^{-1} - 1} \tag{S35}$$

and pick a rate

$$r = \frac{D^\infty_{\mathbb{F}}(\rho)}{D^\infty_{\mathbb{F}}(\omega)} - \chi \tag{S36}$$

for some $\chi > 0$. For all $\xi, \xi' > 0$ and for all large enough n, it then holds that

$$\begin{aligned} \log\lambda_n &\le rn\, D^\infty_{\mathbb{F}}(\omega) + n\xi \\ &\le n\, D^\infty_{\mathbb{F}}(\rho) - n\chi D^\infty_{\mathbb{F}}(\rho) + n\xi \end{aligned} \tag{S37}$$

and

$$\log a_n^{-1} \ge n\, D^\infty_{\mathbb{F}}(\rho) - n\xi'. \tag{S38}$$

Picking $\xi' = \xi = \chi D^\infty_{\mathbb{F}}(\rho)/4$, we get

$$\begin{aligned} \lim_{n\to\infty}\mu_n &= \lim_{n\to\infty}\frac{\lambda_n - 1}{a_n^{-1} - 1} \\ &\le \lim_{n\to\infty}\frac{2^{nD^\infty_{\mathbb{F}}(\rho)(1-\chi+\chi/4)} - 1}{2^{nD^\infty_{\mathbb{F}}(\rho)(1-\chi/4)} - 1} \\ &= \lim_{n\to\infty}\frac{2^{nD^\infty_{\mathbb{F}}(\rho)(1-\chi/4) - nD^\infty_{\mathbb{F}}(\rho)\chi/2} - 1}{2^{nD^\infty_{\mathbb{F}}(\rho)(1-\chi/4)} - 1} \\ &= \lim_{n\to\infty} 2^{-nD^\infty_{\mathbb{F}}(\rho)\chi/2} \\ &= 0. \end{aligned} \tag{S39}$$

Altogether, the probabilistic protocol $(\mathcal{E}_n)_n$ with the choice of μ_n as in Eq. (S35) performs the transformation $\rho \to \omega$ at the rate $\frac{D^\infty_{\mathbb{F}}(\rho)}{D^\infty_{\mathbb{F}}(\omega)} - \chi$ with error satisfying

$$\begin{aligned} \limsup_{n\to\infty} 1 - F\left(\frac{\mathcal{E}_n(\rho^{\otimes n})}{\operatorname{Tr}\mathcal{E}_n(\rho^{\otimes n})}, \omega^{\otimes\lfloor rn\rfloor}\right) &\le 1 - \liminf_{n\to\infty}\frac{1 - \delta_n}{1 - \delta_n + \mu_n\delta_n}(1 - \varepsilon_n) \\ &= 1 - \liminf_{n\to\infty}\left(1 + \frac{\mu_n\delta_n}{1 - \delta_n}\right)^{-1}(1 - \varepsilon_n) \\ &= 0 \end{aligned} \tag{S40}$$

and probability of success

$$\begin{aligned}\liminf_{n\to\infty} p_n &\geq 1 - \limsup_{n\to\infty} \delta_n (1-\mu_n) \\ &= 1-\delta \\ &> 0.\end{aligned} \tag{S41}$$

Since χ was arbitrary, any rate below $\frac{D^\infty_{\mathbb{F}}(\rho)}{D^\infty_{\mathbb{F}}(\omega)}$ is thus achievable, and the result follows. ■

Lemma S3 (Probabilistic monotonicity of max-relative entropy). *For any probabilistic operation $\mathcal{E} \in \mathbb{O}_{\mathrm{RNG},\delta}$ and any $\eta < \operatorname{Tr} \mathcal{E}(\rho)$, it holds that*

$$D^\eta_{\max,\mathbb{F}}(\rho) \geq D^{\eta/\operatorname{Tr}\mathcal{E}(\rho)}_{\max,\mathbb{F}}\left(\frac{\mathcal{E}(\rho)}{\operatorname{Tr}\mathcal{E}(\rho)}\right) + \log\left(\operatorname{Tr}\mathcal{E}(\rho) - \eta\right) - \log(1+\delta). \tag{S42}$$

Proof. Consider any state ρ' with $\frac{1}{2}\left\|\rho-\rho'\right\|_1 \leq \eta$. The first step is to leverage the strong monotonicity of the generalised robustness [14]. Explicitly, write $2^{D_{\max,\mathbb{F}}(\rho')} = \inf\left\{ \lambda \geq 1 \;\middle|\; \rho' \leq \lambda\sigma,\ \sigma \in \mathbb{F}\right\}$ and let $\sigma \in \mathbb{F}$ be any feasible solution to this problem. Then

$$\begin{aligned}\frac{\mathcal{E}(\rho')}{\operatorname{Tr}\mathcal{E}(\rho')} &\leq \lambda\, \frac{\mathcal{E}(\sigma)}{\operatorname{Tr}\mathcal{E}(\rho')} \\ &= \lambda\, \frac{\operatorname{Tr}\mathcal{E}(\sigma)}{\operatorname{Tr}\mathcal{E}(\rho')}\, \frac{\mathcal{E}(\sigma)}{\operatorname{Tr}\mathcal{E}(\sigma)} \\ &\leq \lambda\,(1+\delta)\, \frac{\operatorname{Tr}\mathcal{E}(\sigma)}{\operatorname{Tr}\mathcal{E}(\rho')}\, \sigma' \\ &\leq \lambda\,(1+\delta)\, \frac{1}{\operatorname{Tr}\mathcal{E}(\rho')}\, \sigma',\end{aligned} \tag{S43}$$

where we used the fact that $\mathcal{E}$ is a positive and trace–non-increasing map, as well as that $\frac{\mathcal{E}(\sigma)}{\operatorname{Tr}\mathcal{E}(\sigma)} \leq (1+\delta)\sigma'$ for some $\sigma' \in \mathbb{F}$ by definition of $\mathbb{O}_{\mathrm{RNG},\delta}$. Optimising over all feasible λ gives

$$D_{\max}\left(\frac{\mathcal{E}(\rho')}{\operatorname{Tr}\mathcal{E}(\rho')}\right) \leq D_{\max,\mathbb{F}}(\rho') - \log\operatorname{Tr}\mathcal{E}(\rho') + \log(1+\delta). \tag{S44}$$

Now, since $\frac{1}{2}\left\|\rho-\rho'\right\|_1 \leq \eta$, we have $\left|\operatorname{Tr}\mathcal{E}(\rho) - \operatorname{Tr}\mathcal{E}(\rho')\right| \leq \eta$, so we can bound $\operatorname{Tr}\mathcal{E}(\rho') \geq \operatorname{Tr}\mathcal{E}(\rho) - \eta$. Furthermore, for any two states and any trace non-increasing positive map $\mathcal{E}$ an application of the data processing inequality for the generalised trace distance [15, Proposition 3.8] shows that [16, proof of Proposition 6]

$$\begin{aligned}\frac{1}{2}\left\|\frac{\mathcal{E}(\rho)}{\operatorname{Tr}\mathcal{E}(\rho)} - \frac{\mathcal{E}(\rho')}{\operatorname{Tr}\mathcal{E}(\rho')}\right\|_1 &\leq \frac{1}{2}\left\|\frac{\mathcal{E}(\rho)}{\operatorname{Tr}\mathcal{E}(\rho)} - \frac{\mathcal{E}(\rho')}{\operatorname{Tr}\mathcal{E}(\rho)}\right\|_1 + \frac{1}{2}\left\|\frac{\mathcal{E}(\rho')}{\operatorname{Tr}\mathcal{E}(\rho)} - \frac{\mathcal{E}(\rho')}{\operatorname{Tr}\mathcal{E}(\rho')}\right\|_1 \\ &= \frac{1}{2}\left\|\frac{\mathcal{E}(\rho) - \mathcal{E}(\rho')}{\operatorname{Tr}\mathcal{E}(\rho)}\right\|_1 + \frac{1}{2}\left\|\frac{\mathcal{E}(\rho')\operatorname{Tr}\mathcal{E}(\rho') - \mathcal{E}(\rho')\operatorname{Tr}\mathcal{E}(\rho)}{\operatorname{Tr}\mathcal{E}(\rho)\operatorname{Tr}\mathcal{E}(\rho')}\right\|_1 \\ &= \frac{1}{2}\left\|\frac{\mathcal{E}(\rho) - \mathcal{E}(\rho')}{\operatorname{Tr}\mathcal{E}(\rho)}\right\|_1 + \frac{1}{2}\left|\frac{\operatorname{Tr}\mathcal{E}(\rho') - \operatorname{Tr}\mathcal{E}(\rho)}{\operatorname{Tr}\mathcal{E}(\rho)}\right| \\ &\leq \frac{\frac{1}{2}\left\|\rho-\rho'\right\|_1}{\operatorname{Tr}\mathcal{E}(\rho)},\end{aligned} \tag{S45}$$

which altogether gives

$$D^{\eta/\operatorname{Tr}\mathcal{E}(\rho)}_{\max,\mathbb{F}}\left(\frac{\mathcal{E}(\rho)}{\operatorname{Tr}\mathcal{E}(\rho)}\right) \leq D_{\max,\mathbb{F}}\left(\frac{\mathcal{E}(\rho')}{\operatorname{Tr}\mathcal{E}(\rho')}\right) \leq D_{\max,\mathbb{F}}(\rho') - \log(\operatorname{Tr}\mathcal{E}(\rho) - \eta) + \log(1+\delta). \tag{S46}$$

Optimising over all feasible ρ' yields the statement of the Lemma. ■

ENTANGLEMENT THEORY

Our results in the subsequent sections will specialise to the theory of entanglement. We use SEP to refer to the separable states, i.e. the free states of this resource theory. Resource–non-generating operations $\mathbb{O}_{\text{RNG}}$ correspond here to non-entangling operations NE, and asymptotically resource–non-generating operations $\mathbb{O}_{\text{ARNG}}$ to asymptotically non-entangling operations ANE.

III. EQUIVALENCE OF STRONG CONVERSE AND PROBABILISTIC DISTILLATION IN ENTANGLEMENT THEORY

Recall that $E_{d,\mathbb{O}}^{p=1}(\rho) := r_{p=1}(\rho \to \Phi_+)$, $E_{d,\mathbb{O}}^{p=1,\dagger}(\rho) := r_{p=1}^{\dagger}(\rho \to \Phi_+)$, and $E_{d,\mathbb{O}}^{p>0}(\rho) := r_{p>0}(\rho \to \Phi_+)$.

In order to study transformations of entangled states under different free operations in entanglement theory, in particular LOCC, it is important to note that now it does not suffice to study sub-normalised quantum operations $\mathcal{E}$ like we did previously for RNG operations, as we must ensure that the overall instrument $(\mathcal{E}, \mathcal{E}')$ can be realised as a free protocol. To simplify the considerations, we make two observations:

(i) Let $\mathbb{O}$ be any class of operations that includes LOCC. Due to isotropic twirling [17], the output of any distillation protocol with $\mathbb{O}$ can be assumed without loss of generality to be of the form

$$\varsigma(m, \varepsilon) := (1-\varepsilon)\,\Phi_2^{\otimes m} + \varepsilon \tau_{2^m}, \tag{S47}$$

where

$$\Phi_d := |\Phi_d\rangle\langle\Phi_d| \,, \qquad |\Phi_d\rangle := \frac{1}{\sqrt{d}} \sum_{i=0}^{d-1} |ii\rangle \,, \qquad \tau_d := \frac{\mathbb{1} - \Phi_d}{d^2 - 1} \,. \tag{S48}$$

Now, given a positive integer $m \in \mathbb{N}$ representing the number of local qubits and two error probabilities $\xi, \delta \in [0,1]$ such that $\xi + \delta \le 1$, let us define the $(2^m+1) \times (2^m+1)$ bipartite state

$$\begin{aligned} \omega(m, \xi, \delta) &:= (1 - \xi - \delta)\,\Phi_2^{\otimes m} + \xi\, \tau_{2^m} + \delta\, |ee\rangle\langle ee| \\ &= \left(1 - \frac{\xi}{1-4^{-m}} - \delta\right) \Phi_2^{\otimes m} + \frac{\xi}{1-4^{-m}} \left(\frac{\mathbb{1}}{4}\right)^{\otimes m} + \delta\, |ee\rangle\langle ee| \,, \end{aligned} \tag{S49}$$

where $|e\rangle$ represents a local error flag that is orthogonal to the Hilbert space corresponding to the m qubits.

(ii) Instead of probabilistic transformations into isotropic states as in (S47), we can then consider deterministic transformations into $\omega(m, \xi, q)$. This is because, on the one hand, for any probabilistic distillation protocol which results in the state $\varsigma(m, \varepsilon)$ with some probability p, if the protocol fails, Alice and Bob can simply prepare the state $|ee\rangle\langle ee|$ with probability $1 - p$, thus obtaining the state $\omega(m, \varepsilon p, 1 - p)$ deterministically. On the other hand, any deterministic protocol which results in the state $\omega(m, \xi, \delta)$ can be easily modified to yield the state $\varsigma(m, \xi/(1-\delta))$ with probability $1 - \delta$ by simply measuring whether an error occurred or not. In particular, for any non-vanishing p, the error ε vanishes in the limit $m \to \infty$ if and only if so does ξ.

Let $\mathbb{O}$ be a class of free operations in entanglement theory that includes LOCC. Given as above $\xi, \delta \in [0,1)$ such that $\xi + \delta < 1$, let us define the corresponding one-shot probabilistically distillable entanglement by

$$E_{d,\mathbb{O}}^{(1),\xi,\delta}(\rho) := \max\left\{ m \in \mathbb{N} \;\middle|\; \exists\, \mathcal{E} \in \mathbb{O} \cap \text{CPTP}:\ \mathcal{E}(\rho) = \omega(m, \xi, \delta) \right\}, \tag{S50}$$

where ρ is a generic state, and $\omega(m, \xi, \delta)$ is defined by (S49). Asymptotically, we can set

$$E_{d,\mathbb{O}}^{\xi,\delta}(\rho) := \liminf_{n\to\infty} \frac{1}{n} E_{d,\mathbb{O}}^{(1),\xi,\delta}(\rho^{\otimes n}). \tag{S51}$$

Lemma S4. *Let $m \in \mathbb{N}$ be a positive integer, and consider $\xi, \delta \in [0,1]$ with $\xi + \delta \le 1$. Then, for all*

$\lambda \in [0,1]$ and all $k \in \mathbb{N}$ with $k < m$ the following transitions are possible via LOCC:

$$\omega(m,\xi,\delta) \to \omega(m',\xi',\delta')\,, \qquad m' = m\,, \qquad \xi' = \xi + \lambda\delta(1-2^{-m})\,, \quad \delta' = \delta(1-\lambda)\,; \tag{S52}$$

$$\omega(m,\xi,\delta) \to \omega(m'',\xi'',\delta'')\,, \qquad m'' = m-k\,, \quad \xi'' = \frac{1-4^{-m+k}}{1-4^{-m}}\,\frac{\xi}{2^k}\,, \qquad \delta'' = \delta + \frac{1-2^{-k}}{1-4^{-m}}\,\xi\,. \tag{S53}$$

Proof. The transformation of type (S52) is obtained by the following procedure:

(i) Alice and Bob check if the system is in the error state $|e\rangle$; if it is not, they do nothing;

(ii) if it is, with some probability λ they prepare the separable [17] state $2^{-m}\Phi_2^{\otimes m} + (1-2^{-m})\tau_{2^m}$; with probability $1-\lambda$, they do nothing.

The transformation of type (S53), instead, is obtained by a different procedure. Intuitively, we sacrifice k pairs of qubits to try and determine whether the system is in the maximally entangled state or nor. More precisely, we run the following protocol:

(i) Alice and Bob check if the system is in the error state $|e\rangle$; if it is, they do nothing;

(ii) if it is not, then they measure the first r qubits in the computational basis;

(iii) if upon communicating the outcomes they find that some of them differ, they declare an error, re-preparing the rest of the system in the state $|ee\rangle$;

(iv) if instead all outcomes are found to coincide pairwise, they do nothing on the remaining $m-k$ pairs of qubits.

The elementary computations needed to verify that these protocols yield the claimed transformations are left to the reader. ∎

Proposition S5. *Let $\mathbb{O}$ be a class of free operations in entanglement theory that is closed under composition with LOCCs. Then, for a fixed ρ, the distillable entanglement $E_{d,\mathbb{O}}^{\xi,\delta}(\rho)$ depends on $\xi,\delta \in [0,1)$ only through the sum $\xi+\delta$, or equivalently through $p(1-\varepsilon)$.*

Proof. For asymptotically large m and fixed k, transformations (S52)–(S53) simplify considerably, becoming

$$\omega(m,\xi,\delta) \to \omega(m',\xi',\delta')\,, \qquad m' = m\,, \qquad \xi' \approx \xi + \lambda\delta\,, \quad \delta' = \delta(1-\lambda)\,; \tag{S54}$$

$$\omega(m,\xi,\delta) \to \omega(m'',\xi'',\delta'')\,, \qquad m'' = m-k\,, \quad \xi'' \approx \frac{\xi}{2^k}\,, \qquad \delta'' \approx \delta + (1-2^{-k})\,\xi\,. \tag{S55}$$

With these transformations, it is clear that we can turn a pair (ξ,δ) with $\xi+\delta<1$ into another pair (ξ',δ') with $\xi'+\delta'<1$ if and only if $\xi+\delta = \xi'+\delta'$. ∎

A corollary of the above is Theorem 3 in the main text, whose extended statement we present here.

Theorem 3. *Let $\mathbb{O}$ be any class of operations which is closed under composition with LOCC, i.e. such that $\mathcal{E}\in\mathbb{O}$, $\mathcal{F}\in$ LOCC $\Rightarrow$ $\mathcal{F}\circ\mathcal{E}\in\mathbb{O}$. This includes in particular the set LOCC itself.*
Consider then any sequence of entanglement transformation protocols $(\mathcal{E}_n)_n$ with associated error $\limsup_{n\to\infty}\varepsilon_n = \varepsilon < 1$ and probability of success $\liminf_{n\to\infty} p_n = p > 0$. Then, then there exists another sequence of entanglement distillation protocols $(\mathcal{E}'_n)_n$ with error $\limsup_{n\to\infty}\varepsilon'_n =: \varepsilon'$ and probability $\liminf_{n\to\infty} p'_n =: p'$ if and only if

$$p\,(1-\varepsilon) = p'\,(1-\varepsilon'). \tag{S56}$$

Hence, for all states ρ and all sets of operations $\mathbb{O}$ as above,

$$E_{d,\mathbb{O}}^{p>0}(\rho) = E_{d,\mathbb{O}}^{p=1,\dagger}(\rho) = E_{d,\mathbb{O}}^{p>0,\dagger}(\rho). \tag{S57}$$

In particular, for all non-entangling operations it holds that

$$\begin{aligned} D^{\infty}_{\mathrm{SEP}}(\rho) &= E^{p>0}_{d,\mathrm{NE}}(\rho) = E^{p=1,\dagger}_{d,\mathrm{NE}}(\rho) = E^{p=1,\ddagger}_{d,\mathrm{NE}}(\rho) \\ &= E^{p>0}_{d,\mathrm{ANE}}(\rho) = E^{p=1,\dagger}_{d,\mathrm{ANE}}(\rho) = E^{p=1,\ddagger}_{d,\mathrm{ANE}}(\rho). \end{aligned} \tag{S58}$$

The last statement in the above can be seen from the result that $D^{\infty}_{\mathrm{SEP}}(\rho) = E^{p=1,\dagger}_{d,\mathrm{NE}}(\rho) = E^{p=1,\ddagger}_{d,\mathrm{ANE}}(\rho)$ [9] (cf. [1, Corollary III.3]), where the latter term denotes the strong converse distillable entanglement defined using the slightly stronger notion of a strong converse rate found in Eq. (S7).[3]

A more direct proof of the fact that the rate $D^{\infty}_{\mathrm{SEP}}(\rho)$ can be achieved by probabilistic non-entangling operations (as opposed to *asymptotically* non-entangling ones as in Theorem 1) can be obtained by following the proof of Theorem 1 but choosing $\omega_n = \Phi_2^{\otimes \lfloor rn \rfloor}$ and realising that we can then pick π_n to be separable states [6, 18], so no resources need to be generated whatsoever.

IV. IRREVERSIBILITY OF ENTANGLEMENT THEORY UNDER PROBABILISTIC NON-ENTANGLING OPERATIONS

Although here we will focus on (asymptotically) non-entangling operations (A)NE, i.e. (A)RNG maps with the set of free states $\mathbb{F} = \mathrm{SEP}$, the discussion below applies verbatim also to the case of $\mathbb{F} = \mathrm{PPT}$, which corresponds to (asymptotically) PPT–non-generating operations. This in particular implies irreversibility under PPT operations [19] enhanced by asymptotic entanglement non-generation.

Recall that we defined asymptotically resource–non-generating operations (and hence also asymptotically non-entangling ones, ANE) using the generalised robustness $R^{g}_{\mathbb{F}}$ (see Eq. (S10)). Let us consider two amended variants of this definition: one defined using the *standard robustness* [6]

$$R^{s}_{\mathrm{SEP}}(\rho) := \inf\left\{ \lambda \in \mathbb{R}_+ \;\middle|\; \frac{\rho + \lambda\sigma}{1+\lambda} \in \mathrm{SEP},\; \sigma \in \mathrm{SEP} \right\}, \tag{S59}$$

and one using the *negativity* [20]

$$N(\rho) := \frac{1}{2}\left(\left\| \rho^{\Gamma} \right\|_1 - 1 \right) \tag{S60}$$

with Γ denoting partial transpose. The classes of operations are

$$\begin{aligned} \mathbb{O}_{\mathrm{NE},\delta,s} &:= \left\{ \mathcal{E} \in \mathrm{CPTNI} \;\middle|\; R^{s}_{\mathrm{SEP}}\left(\frac{\mathcal{E}(\sigma)}{\operatorname{Tr}\mathcal{E}(\sigma)} \right) \le \delta \;\forall \sigma \in \mathrm{SEP} \right\}, \\ \mathbb{O}_{\mathrm{NE},\delta,N} &:= \left\{ \mathcal{E} \in \mathrm{CPTNI} \;\middle|\; N\left(\frac{\mathcal{E}(\sigma)}{\operatorname{Tr}\mathcal{E}(\sigma)} \right) \le \delta \;\forall \sigma \in \mathrm{SEP} \right\}. \end{aligned} \tag{S61}$$

The corresponding asymptotic transformation rates are defined analogously as before,

$$\begin{aligned} r_{p>0}(\rho \xrightarrow[\mathrm{ANE},s]{} \omega) := \sup_{(\mathcal{E}_n)_n} \Bigg\{ r \;\Bigg|\; & \lim_{n\to\infty} F\left(\frac{\mathcal{E}_n(\rho^{\otimes n})}{\operatorname{Tr}\mathcal{E}_n(\rho^{\otimes n})}, \omega^{\otimes \lfloor rn \rfloor} \right) = 1, \\ & \lim_{n\to\infty} \sup_{\sigma\in\mathrm{SEP}} R^{s}_{\mathrm{SEP}}\left(\frac{\mathcal{E}_n(\sigma)}{\operatorname{Tr}\mathcal{E}_n(\sigma)} \right) = 0, \quad \liminf_{n\to\infty} \operatorname{Tr}\mathcal{E}_n(\rho^{\otimes n}) > 0 \Bigg\}, \\ r_{p>0}(\rho \xrightarrow[\mathrm{ANE},N]{} \omega) := \sup_{(\mathcal{E}_n)_n} \Bigg\{ r \;\Bigg|\; & \lim_{n\to\infty} F\left(\frac{\mathcal{E}_n(\rho^{\otimes n})}{\operatorname{Tr}\mathcal{E}_n(\rho^{\otimes n})}, \omega^{\otimes \lfloor rn \rfloor} \right) = 1, \\ & \lim_{n\to\infty} \sup_{\sigma\in\mathrm{SEP}} N\left(\frac{\mathcal{E}_n(\sigma)}{\operatorname{Tr}\mathcal{E}_n(\sigma)} \right) = 0, \quad \liminf_{n\to\infty} \operatorname{Tr}\mathcal{E}_n(\rho^{\otimes n}) > 0 \Bigg\}. \end{aligned} \tag{S62}$$

---

[3] Whether this equivalence between the two definitions of the strong converse holds also for other sets of free operations $\mathbb{O}$ is an interesting open problem.

Noting that $R^s_{\mathrm{SEP}}(\rho) \geq N(\rho)$ [20], for any state it holds that

$$r_{p>0}(\rho \xrightarrow[\mathrm{NE}]{} \omega) \leq r_{p>0}(\rho \xrightarrow[\mathrm{ANE},s]{} \omega) \leq r_{p>0}(\rho \xrightarrow[\mathrm{ANE},N]{} \omega). \tag{S63}$$

That is, imposing the asymptotic vanishing of the negativity is the weakest of the constraints that we consider, and thus ANE, N form the most permissive type of transformations. In particular, any transformation that is irreversible under ANE, N is also irreversible under ANE, s and ANE.

As before, we define the probabilistic distillable entanglement $E^{p>0}_{d,\mathrm{ANE},N}(\rho) \coloneqq r_{p>0}(\rho \xrightarrow[\mathrm{ANE},N]{} \Phi_+)$ and $E^{p>0}_{d,\mathrm{ANE},s}(\rho) \coloneqq r_{p>0}(\rho \xrightarrow[\mathrm{ANE},s]{} \Phi_+)$ as well as the probabilistic entanglement cost $E^{p>0}_{c,\mathrm{ANE},N}(\rho) \coloneqq r_{p>0}(\Phi_+ \xrightarrow[\mathrm{ANE},N]{} \rho)^{-1}$ and $E^{p>0}_{c,\mathrm{ANE},s}(\rho) \coloneqq r_{p>0}(\Phi_+ \xrightarrow[\mathrm{ANE},s]{} \rho)^{-1}$.

In [7], it was shown that in the deterministic setting there exists a two-qutrit state ω_3 such that

$$E^{p=1}_{d,\mathrm{ANE},N}(\omega_3) < E^{p=1}_{c,\mathrm{ANE},N}(\omega_3). \tag{S64}$$

Here we generalise this to the probabilistic setting.

Theorem 2. *The state ω_3 satisfies that*

$$E^{p>0}_{d,\mathrm{ANE},N}(\omega_3) = E^{p=1}_{d,\mathrm{ANE},N}(\omega_3) = E^{p=1}_{d,\mathrm{NE}}(\omega_3) < E^{p=1}_{c,\mathrm{NE}}(\omega_3) = E^{p=1}_{c,\mathrm{ANE},N}(\omega_3) = E^{p>0}_{c,\mathrm{ANE},N}(\omega_3). \tag{S65}$$

In particular, even in the probabilistic setting, the theory of entanglement remains irreversible under non-entangling operations, or under operations that generate asymptotically vanishing amounts of entanglement as quantified by the standard robustness R^s_{SEP} or the negativity N.

The same remains true if we replace SEP *with the set* PPT *and* (A)NE *operations with (asymptotically) PPT–non-generating operations.*

The proof of the theorem will rely on two lemmas that establish a characterisation of the probabilistic rates of distillation and dilution, respectively.

Lemma S8 (Relative entropy and equivalence of probabilistic distillation)**.** *For any state ρ, the probabilistic distillable entanglement under* ANE, s *or* ANE, N *operations equals that under* ANE, *that is, it is given by the regularised relative entropy:*

$$E^{p>0}_{d,\mathrm{ANE},s}(\rho) = E^{p>0}_{d,\mathrm{ANE},N}(\rho) = E^{p>0}_{d,\mathrm{ANE}}(\rho) = D^{\infty}_{\mathrm{SEP}}(\rho). \tag{S66}$$

Proof. By the result of Theorem 3, we have that

$$E^{p>0}_{d,\mathrm{ANE},N}(\rho) \geq E^{p>0}_{d,\mathrm{NE}}(\rho) = D^{\infty}_{\mathrm{SEP}}(\rho), \tag{S67}$$

so it remains to show the opposite direction. This can already be seen from Lemma S1 combined with a result of [7]: we have the chain of inequalities

$$E^{p>0}_{d,\mathrm{ANE},N}(\rho) \overset{\text{(i)}}{\leq} E^{p=1,\dagger}_{d,\mathrm{ANE},N}(\rho) \overset{\text{(ii)}}{=} E^{p=1,\dagger}_{d,\mathrm{NE}}(\rho) \overset{\text{(iii)}}{=} D^{\infty}_{\mathrm{SEP}}(\rho)\,, \tag{S68}$$

where (i) follows from Lemma S1, (ii) from [7, Lemma S17], and finally (iii) is known from the works of Brandão and Plenio [1, 9] (see also Theorem 3).

For completeness, we will give a more direct alternative argument. To this end, we would like to establish a converse result as in Theorem 1; however, the probabilistic monotonicity of R^g_{SEP} (Lemma S3) no longer holds when the operations $\mathbb{O}_{\mathrm{NE},\delta,N}$ are considered. Our idea will be to show that an approximate version of Lemma S3 can still be established in the special case of distillation, that is, conversion into the maximally entangled state. This will in fact directly lead to a strong converse bound.

Consider then any sequence $(\mathcal{E}_n)_n$ of operations $\mathcal{E}_n \in \mathbb{O}_{\mathrm{NE},\delta_n,N}$ such that

$$1 - \mathrm{Tr}\left(\frac{\mathcal{E}_n(\rho^{\otimes n})}{\mathrm{Tr}\,\mathcal{E}_n(\rho^{\otimes n})}\,\Phi_+^{\otimes \lfloor rn \rfloor}\right) = 1 - F\left(\frac{\mathcal{E}_n(\rho^{\otimes n})}{\mathrm{Tr}\,\mathcal{E}_n(\rho^{\otimes n})},\,\Phi_+^{\otimes \lfloor rn \rfloor}\right) \eqqcolon \varepsilon_n\,, \tag{S69}$$

with $\liminf_{n\to\infty} \operatorname{Tr}\mathcal{E}_n(\rho^{\otimes n}) := p > 0$. It will be crucial to notice that, for any separable state σ, we have

$$\begin{aligned}\operatorname{Tr}\left(\Phi_+^{\otimes\lfloor rn\rfloor}\mathcal{E}_n(\sigma)\right) &= \operatorname{Tr}\left[\left(\Phi_+^{\otimes\lfloor rn\rfloor}\right)^\Gamma \mathcal{E}_n(\sigma)^\Gamma\right] \\ &\le \operatorname{Tr}\left\|\left(\Phi_+^{\otimes\lfloor rn\rfloor}\right)^\Gamma\right\|_\infty \left\|\mathcal{E}_n(\sigma)^\Gamma\right\|_1 \\ &\le \frac{1}{2^{\lfloor rn\rfloor}}(1+2\delta_n)\operatorname{Tr}\mathcal{E}_n(\sigma) \\ &\le \frac{1}{2^{\lfloor rn\rfloor}}(1+2\delta_n),\end{aligned} \tag{S70}$$

where in the third line we used the fact that the eigenvalues of $\left(\Phi_+^{\otimes\lfloor rn\rfloor}\right)^\Gamma$ — which is proportional to the swap operator — are $\pm 2^{-\lfloor rn\rfloor}$. We further leveraged the fact that

$$\frac{1}{2}\left(\left\|\frac{\mathcal{E}_n(\sigma)^\Gamma}{\operatorname{Tr}\mathcal{E}_n(\sigma)^\Gamma}\right\|_1 - 1\right) \le \delta_n \tag{S71}$$

by the definition of $\mathbb{O}_{\mathrm{NE},\delta_n,N}$, and the last line follows since $\mathcal{E}_n$ is trace non-increasing.

Consider now any error sequence $(\zeta_n)_n$ with $\lim_{n\to\infty}\zeta_n = 0$, so that $\zeta_n < p$ and hence also $\zeta_n < \operatorname{Tr}\mathcal{E}_n(\rho^{\otimes n})$ holds for all sufficiently large n. Let ρ'_n be any state such that $\frac{1}{2}\left\|\rho^{\otimes n} - \rho'_n\right\|_1 \le \zeta_n$, and let $\sigma_n \in \mathrm{SEP}$ be any state such that $\rho'_n \le \lambda_n\sigma_n$ for some λ_n. Then

$$\begin{aligned}1-\varepsilon_n &= \operatorname{Tr}\left(\frac{\mathcal{E}_n(\rho^{\otimes\lfloor rn\rfloor})}{\operatorname{Tr}\mathcal{E}_n(\rho^{\otimes\lfloor rn\rfloor})}\,\Phi_+^{\otimes\lfloor rn\rfloor}\right) \\ &= \operatorname{Tr}\left(\frac{\mathcal{E}_n(\rho'_n)}{\operatorname{Tr}\mathcal{E}_n(\rho'_n)}\,\Phi_+^{\otimes\lfloor rn\rfloor}\right) + \operatorname{Tr}\left(\left[\frac{\mathcal{E}_n(\rho^{\otimes\lfloor rn\rfloor})}{\operatorname{Tr}\mathcal{E}_n(\rho^{\otimes\lfloor rn\rfloor})} - \frac{\mathcal{E}_n(\rho'_n)}{\operatorname{Tr}\mathcal{E}_n(\rho'_n)}\right]\Phi_+^{\otimes\lfloor rn\rfloor}\right) \\ &\le \lambda_n\operatorname{Tr}\left(\frac{\mathcal{E}_n(\sigma_n)}{\operatorname{Tr}\mathcal{E}_n(\rho'_n)}\,\Phi_+^{\otimes\lfloor rn\rfloor}\right) + \frac{1}{2}\left\|\frac{\mathcal{E}_n(\rho^{\otimes\lfloor rn\rfloor})}{\operatorname{Tr}\mathcal{E}_n(\rho^{\otimes\lfloor rn\rfloor})} - \frac{\mathcal{E}_n(\rho'_n)}{\operatorname{Tr}\mathcal{E}_n(\rho'_n)}\right\|_1 \\ &\overset{\text{(iv)}}{\le} \lambda_n\frac{1}{2^{\lfloor rn\rfloor}}(1+2\delta_n)\frac{1}{\operatorname{Tr}\mathcal{E}_n(\rho'_n)} + \frac{\zeta_n}{\operatorname{Tr}\mathcal{E}_n(\rho'_n)} \\ &\le \left(\lambda_n\frac{1}{2^{\lfloor rn\rfloor}}(1+2\delta_n)+\zeta_n\right)\frac{1}{\operatorname{Tr}\mathcal{E}_n(\rho^{\otimes n})-\zeta_n}.\end{aligned} \tag{S72}$$

Here, (iv) follows by Eq. (S70) together with the probabilistic data processing inequality for the trace distance that we previously showed in Eq. (S45).

So far, the above derivation made no assumption about the rate r. Let us now assume that

$$\begin{aligned}r &> D_{\mathrm{SEP}}^\infty(\rho) \\ &= \inf_{(\zeta_n)_n}\left\{\limsup_{n\to\infty}\frac{1}{n}D_{\max,\mathrm{SEP}}^{\zeta_n}(\rho^{\otimes n})\;\middle|\;\lim_{n\to\infty}\zeta_n = 0\right\},\end{aligned} \tag{S73}$$

where the second line follows from the asymptotic equipartition property of Brandão–Plenio–Datta (see [9, Proposition IV.2] [3, Theorem 1]). This implies that there exists an asymptotically vanishing error sequence $(\zeta_n)_n$ such that

$$\limsup_{n\to\infty}\frac{1}{n}D_{\max,\mathrm{SEP}}^{\zeta_n}(\rho^{\otimes n}) < r, \tag{S74}$$

and hence that there exist states ρ'_n with $\frac{1}{2}\left\|\rho^{\otimes n}-\rho'_n\right\|_1 \le \zeta_n$ and $\rho'_n \le \lambda_n\sigma_n$ such that

$$c := r - \frac{1}{n}\log\lambda_n > 0 \tag{S75}$$

for all sufficiently large n. Plugging this into (S72), we have that

$$\begin{aligned}1-\varepsilon_n &\le \left(2^{\log\lambda_n}2^{-rn+1}(1+2\delta_n)+\zeta_n\right)\frac{1}{\operatorname{Tr}\mathcal{E}_n(\rho^{\otimes n})-\zeta_n} \\ &= \left(2^{-cn+1}(1+2\delta_n)+\zeta_n\right)\frac{1}{\operatorname{Tr}\mathcal{E}_n(\rho^{\otimes n})-\zeta_n}.\end{aligned} \tag{S76}$$

Recalling that $\lim_{n\to\infty} \zeta_n = 0$ while $\operatorname{Tr} \mathcal{E}_n(\rho^{\otimes n})$ is lower bounded by a constant, if we furthermore assume that δ_n vanishes in the limit $n \to \infty$ (or indeed even only that $\delta_n = 2^{o(n)}$), the whole term on the right-hand side of Eq. (S76) goes to zero, and we thus obtain

$$\lim_{n\to\infty} \varepsilon_n = 1. \tag{S77}$$

Since this holds for any $r > D^{\infty}_{\mathrm{SEP}}(\rho)$, it means that no rate achievable with error less than one can be larger than $D^{\infty}_{\mathrm{SEP}}$. Therefore

$$E^{p>0}_{d,\mathrm{ANE},N}(\rho) \leq E^{p>0,\ddagger}_{d,\mathrm{ANE},N}(\rho) \leq D^{\infty}_{\mathrm{SEP}}(\rho), \tag{S78}$$

where $E^{p>0,\ddagger}_{d,\mathrm{ANE},N}$ is constructed by using the definition of rate given in Eq. (S9).

■

Our approach to the bounds on entanglement cost will be based on the ideas in [7], where the monotone known as the (logarithmic) *tempered negativity*

$$E_\tau(\rho) := \log \max \left\{ \operatorname{Tr} X\rho \;\middle|\; \left\|X^\Gamma\right\|_\infty \leq 1,\ \|X\|_\infty \leq \operatorname{Tr} X\rho \right\} \tag{S79}$$

was introduced.

Lemma S9 (Restrictions on probabilistic entanglement cost)**.** *For any state ρ, the probabilistic entanglement cost under* ANE, s *and* ANE, N *operations is lower bounded by the tempered negativity:*

$$E^{p>0}_{c,\mathrm{ANE},s}(\rho) \geq E^{p>0}_{c,\mathrm{ANE},N}(\rho) \geq E_\tau(\rho). \tag{S80}$$

For the operations ANE, s, *we can make an even stronger statement: the probabilistic and deterministic entanglement cost are equal, and in fact they both equal the entanglement cost under strictly non-entangling operations:*

$$E^{p>0}_{c,\mathrm{ANE},s}(\rho) = E^{p=1}_{c,\mathrm{ANE},s}(\rho) = E^{p=1}_{c,\mathrm{NE}}(\rho). \tag{S81}$$

Both parts of the lemma rely on a probabilistic monotonicity result for R^s_{SEP}, similar to the one that we previously showed for $R^g_{\mathbb{F}}$ in Lemma S3. We state it as a separate lemma for clarity.

Lemma S10 (Probabilistic monotonicity of standard robustness and negativity)**.** *For any probabilistic operation $\mathcal{E} \in \mathbb{O}_{\mathrm{NE},\delta,N}$, it holds that*

$$1 + R^s_{\mathrm{SEP}}(\rho) \geq \left\| \frac{\mathcal{E}(\rho)^\Gamma}{\operatorname{Tr}\mathcal{E}(\rho)} \right\|_1 \frac{\operatorname{Tr}\mathcal{E}(\rho)}{2(1+2\delta)}. \tag{S82}$$

For any probabilistic operation $\mathcal{E} \in \mathbb{O}_{\mathrm{NE},\delta,s}$, it holds that

$$1 + R^s_{\mathrm{SEP}}(\rho) \geq \left[1 + R^s_{\mathrm{SEP}}\left(\frac{\mathcal{E}(\rho)}{\operatorname{Tr}\mathcal{E}(\rho)} \right) \right] \frac{\operatorname{Tr}\mathcal{E}(\rho)}{1+2\delta}. \tag{S83}$$

Proof. For the case of $\mathcal{E} \in \mathbb{O}_{\mathrm{NE},\delta,N}$, consider any feasible solution for $R^s_{\mathrm{SEP}}(\rho)$, that is, a decomposition $\rho = (1+\lambda)\,\sigma_+ - \lambda\sigma_-$ with $\sigma_\pm \in \mathrm{SEP}$. Then

$$\begin{aligned}
\left\| \frac{\mathcal{E}(\rho)^\Gamma}{\operatorname{Tr}\mathcal{E}(\rho)} \right\|_1 &\leq (1+\lambda) \frac{\operatorname{Tr}\mathcal{E}(\sigma_+)}{\operatorname{Tr}\mathcal{E}(\rho)} \left\| \frac{\mathcal{E}(\sigma_+)^\Gamma}{\operatorname{Tr}\mathcal{E}(\sigma_+)} \right\|_1 + \lambda \frac{\operatorname{Tr}\mathcal{E}(\sigma_-)}{\operatorname{Tr}\mathcal{E}(\rho)} \left\| \frac{\mathcal{E}(\sigma_-)^\Gamma}{\operatorname{Tr}\mathcal{E}(\sigma_-)} \right\|_1 \\
&\leq \left[(1+\lambda) \frac{\operatorname{Tr}\mathcal{E}(\sigma_+)}{\operatorname{Tr}\mathcal{E}(\rho)} + \lambda \frac{\operatorname{Tr}\mathcal{E}(\sigma_-)}{\operatorname{Tr}\mathcal{E}(\rho)} \right] (1+2\delta) \\
&\leq \frac{1+2\lambda}{\operatorname{Tr}\mathcal{E}(\rho)} (1+2\delta) \\
&\leq 2 \frac{1+\lambda}{\operatorname{Tr}\mathcal{E}(\rho)} (1+2\delta),
\end{aligned} \tag{S84}$$

where in the second line we used that $N\left(\frac{\mathcal{E}(\sigma_\pm)}{\operatorname{Tr}\mathcal{E}(\sigma_\pm)}\right) \leq \delta$ by definition of $\mathbb{O}_{\mathrm{NE},\delta,N}$, and in the third line we used that the operation $\mathcal{E}$ may not increase trace. Optimising over all feasible λ we get the stated result.

For $\mathbb{O}_{\mathrm{NE},\delta,s}$, we will use three equivalent definitions of R^s_{SEP}, all of which follow straightforwardly from the original definition by using the fact that $\operatorname{Tr}\rho = 1$:

$$\begin{aligned} R^s_{\mathrm{SEP}}(\rho) &= \min\left\{\lambda \;\middle|\; \rho = (1+\lambda)\,\sigma_+ - \lambda\sigma_-,\ \sigma_\pm \in \mathrm{SEP}\right\} \\ &= \min\left\{\lambda \;\middle|\; \rho \leq_{\mathrm{SEP}} (1+\lambda)\sigma_+,\ \sigma_+ \in \mathrm{SEP}\right\} \\ &= \min\left\{\lambda \;\middle|\; \rho \geq_{\mathrm{SEP}} -\lambda\sigma_-,\ \sigma_- \in \mathrm{SEP}\right\}, \end{aligned} \tag{S85}$$

where $\leq_{\mathrm{SEP}}$ denotes inequality with respect to the cone of separable operators. Let $\rho = (1+\lambda)\,\sigma_+ - \lambda\sigma_-$ be any feasible decomposition for ρ. Then, for any $\mathcal{E} \in \mathbb{O}_{\mathrm{NE},\delta,s}$, it holds that

$$\begin{aligned} \frac{\mathcal{E}(\rho)}{\operatorname{Tr}\mathcal{E}(\rho)} &= (1+\lambda)\frac{\operatorname{Tr}\mathcal{E}(\sigma_+)}{\operatorname{Tr}\mathcal{E}(\rho)}\frac{\mathcal{E}(\sigma_+)}{\operatorname{Tr}\mathcal{E}(\sigma_+)} - \lambda\frac{\operatorname{Tr}\mathcal{E}(\sigma_-)}{\operatorname{Tr}\mathcal{E}(\rho)}\frac{\mathcal{E}(\sigma_-)}{\operatorname{Tr}\mathcal{E}(\sigma_-)} \\ &\leq_{\mathrm{SEP}} (1+\lambda)\frac{\operatorname{Tr}\mathcal{E}(\sigma_+)}{\operatorname{Tr}\mathcal{E}(\rho)}(1+\delta)\,\sigma'_+ + \lambda\frac{\operatorname{Tr}\mathcal{E}(\sigma_-)}{\operatorname{Tr}\mathcal{E}(\rho)}\delta\,\sigma'_- \end{aligned} \tag{S86}$$

for some $\sigma'_\pm \in \mathrm{SEP}$, where we used that $\mathcal{E}$ can only generate at most δ robustness from any separable state. Since the operator in the last line is a non-negative combination of separable operators it is separable itself, and therefore it constitutes a feasible solution for the robustness of $\frac{\mathcal{E}(\rho)}{\operatorname{Tr}\mathcal{E}(\rho)}$. We can find its normalisation by simply taking the trace. Thus

$$\begin{aligned} 1 + R^s_{\mathrm{SEP}}\left(\frac{\mathcal{E}(\rho)}{\operatorname{Tr}\mathcal{E}(\rho)}\right) &\leq \frac{(1+\lambda)(1+\delta)\ \operatorname{Tr}\mathcal{E}(\sigma_+) + \lambda\delta\ \operatorname{Tr}\mathcal{E}(\sigma_-)}{\operatorname{Tr}\mathcal{E}(\rho)} \\ &\leq \frac{(1+\lambda)(1+\delta) + \lambda\delta}{\operatorname{Tr}\mathcal{E}(\rho)} \\ &\leq \frac{(1+\lambda)(1+\delta) + (1+\lambda)\delta}{\operatorname{Tr}\mathcal{E}(\rho)} \\ &= \frac{(1+\lambda)(1+2\delta)}{\operatorname{Tr}\mathcal{E}(\rho)} \end{aligned} \tag{S87}$$

using the trace–non-increasing property of $\mathcal{E}$. ■

Proof of Lemma S9. Consider first ANE, N cost. Let $(\mathcal{E}_n)_n$ be any sequence of operations $\mathcal{E}_n \in \mathbb{O}_{\mathrm{NE},\delta_n,N}$ such that $\frac{1}{2}\left\|\frac{\mathcal{E}_n(\Phi_+^{\otimes n})}{\operatorname{Tr}\mathcal{E}_n(\Phi_+^{\otimes n})} - \rho^{\otimes\lfloor rn\rfloor}\right\|_1 =: \varepsilon_n$. Using that $R^s_{\mathrm{SEP}}(\Phi_+^{\otimes n}) = 2^n - 1$ [6], we have

$$\begin{aligned} n &= \log\left(1 + R^s_{\mathrm{SEP}}(\Phi_+^{\otimes n})\right) \\ &\overset{\text{(i)}}{\geq} \log\left\|\frac{\mathcal{E}_n(\Phi_+^{\otimes n})^\Gamma}{\operatorname{Tr}\mathcal{E}_n(\Phi_+^{\otimes n})}\right\|_1 + \log\operatorname{Tr}\mathcal{E}_n(\Phi_+^{\otimes n}) - \log 2 - \log(1+2\delta_n) \\ &\overset{\text{(ii)}}{\geq} E_\tau\left(\rho^{\otimes\lfloor rn\rfloor}\right) + \log(1-2\varepsilon_n) + \log\operatorname{Tr}\mathcal{E}_n(\Phi_+^{\otimes n}) - \log 2 - \log(1+2\delta_n) \\ &\overset{\text{(iii)}}{\geq} \lfloor rn\rfloor\, E_\tau(\rho) + \log(1-2\varepsilon_n) + \log\operatorname{Tr}\mathcal{E}_n(\Phi_+^{\otimes n}) - \log 2 - \log(1+2\delta_n), \end{aligned} \tag{S88}$$

where: (i) follows from Lemma S10; (ii) follows from Proposition S5 and Lemma S6 of [7] (cf. proofs of Theorems 1 and S16 therein); (iii) follows from the super-additivity of E_τ, shown in Proposition S5 of [7]. Assuming that $\operatorname{Tr}\mathcal{E}_n(\Phi_+^{\otimes n}) = 2^{-o(n)}$, $\delta_n = 2^{o(n)}$ and $\limsup_n \varepsilon_n < \frac{1}{2}$ (which are all, in fact, weaker than our assumptions) we can divide by n and take the lim inf as $n \to \infty$ to get

$$r^{-1} \geq E_\tau(\rho), \tag{S89}$$

which is what was to be shown.

For the case of ANE, s, it is immediate from the definitions that $E^{p>0}_{c,\mathrm{ANE},s}(\rho) \leq E^{p=1}_{c,\mathrm{NE}}(\rho)$, so we need to show the opposite inequality. We know from the results of Brandão and Plenio [9, Sec. V] that

$$E^{p=1}_{c,\mathrm{NE}}(\rho) = \inf_{(\zeta_n)_n}\left\{\limsup_{n\to\infty}\frac{1}{n}\log\left[1 + R^{s,\zeta_n}_{\mathrm{SEP}}\left(\rho^{\otimes n}\right)\right] \;\middle|\; \lim_{n\to\infty}\zeta_n = 0\right\}, \tag{S90}$$

where we denoted

$$R^{s,\zeta}_{\mathrm{SEP}}(\rho) := \min_{\frac{1}{2}\|\rho'-\rho\|_1 \leq \zeta} R^s_{\mathrm{SEP}}(\rho'). \tag{S91}$$

Although an exact expression for the quantity on the right-hand side of (S90) is not known (unlike in the asymptotic equipartition property for R^g_{SEP} that we used earlier), we can still use it to establish a converse bound. Take any sequence of operations $(\mathcal{E}_n)_n$ with $\mathcal{E}_n \in \mathbb{O}_{\mathrm{NE},\delta_n,s}$ such that $\frac{1}{2}\left\|\frac{\mathcal{E}_n(\Phi_+^{\otimes n})}{\operatorname{Tr}\mathcal{E}_n(\Phi_+^{\otimes n})} - \rho^{\otimes\lfloor rn\rfloor}\right\|_1 =: \varepsilon_n$ with $\liminf_n \operatorname{Tr}\mathcal{E}_n(\Phi_+^{\otimes n}) > 0$ and $\lim_n \varepsilon_n = 0$. We then have that

$$\begin{aligned} n &= \log\left(1 + R^s_{\mathrm{SEP}}(\Phi_+^{\otimes n})\right) \\ &\geq \log\left(1 + R^s_{\mathrm{SEP}}\left(\frac{\mathcal{E}_n(\Phi_+^{\otimes n})}{\operatorname{Tr}\mathcal{E}_n(\Phi_+^{\otimes n})}\right)\right) + \log\operatorname{Tr}\mathcal{E}_n(\Phi_+^{\otimes n}) - \log(1+2\delta_n) \\ &\geq \log\left(1 + R^{s,\varepsilon_n}_{\mathrm{SEP}}\left(\rho^{\otimes\lfloor rn\rfloor}\right)\right) + \log\operatorname{Tr}\mathcal{E}_n(\Phi_+^{\otimes n}) - \log(1+2\delta_n) \end{aligned} \tag{S92}$$

using Lemma S10. As before, the two rightmost terms will vanish asymptotically provided that $\operatorname{Tr}\mathcal{E}_n(\Phi_+^{\otimes n}) = 2^{-o(n)}$ and $\delta_n = 2^{o(n)}$. Dividing by n, taking the lim sup, and using that $\lim_{n\to\infty}\varepsilon_n = 0$ gives

$$1 \geq \inf_{(\zeta_n)_n}\left\{\limsup_{n\to\infty}\frac{1}{n}\log\left[1 + R^{s,\zeta_n}_{\mathrm{SEP}}\left(\rho^{\otimes\lfloor rn\rfloor}\right)\right] \;\middle|\; \lim_{n\to\infty}\zeta_n = 0\right\}. \tag{S93}$$

To argue that $r^{-1} \geq E^{p=1}_{c,\mathrm{NE}}(\rho)$, it thus remains to show that

$$\begin{aligned} &\inf_{(\zeta_n)_n}\left\{\limsup_{n\to\infty}\frac{1}{n}\log\left[1 + R^{s,\zeta_n}_{\mathrm{SEP}}\left(\rho^{\otimes\lfloor rn\rfloor}\right)\right] \;\middle|\; \lim_{n\to\infty}\zeta_n = 0\right\} \\ &\geq r\inf_{(\zeta_n)_n}\left\{\limsup_{n\to\infty}\frac{1}{n}\log\left[1 + R^{s,\zeta_n}_{\mathrm{SEP}}\left(\rho^{\otimes n}\right)\right] \;\middle|\; \lim_{n\to\infty}\zeta_n = 0\right\} \\ &= r\,E^{p=1}_{c,\mathrm{NE}}(\rho). \end{aligned} \tag{S94}$$

To see this, let us begin by defining for any $n \in \mathbb{N}$ the corresponding quantity

$$n^{(r)} := \min\left\{\lfloor rl\rfloor \;\middle|\; l \in \mathbb{N},\ \lfloor rl\rfloor \geq n\right\}. \tag{S95}$$

For any fixed asymptotically vanishing error sequence $(\zeta_n)_n$, we also define

$$\zeta^{(r)}_n := \max\left\{\zeta_l \;\middle|\; l \in \mathbb{N},\ \lfloor rl\rfloor \geq n\right\}. \tag{S96}$$

Then

$$\begin{aligned} \limsup_{n\to\infty}\frac{1}{n}\log\left[1 + R^{s,\zeta_n}_{\mathrm{SEP}}\left(\rho^{\otimes\lfloor rn\rfloor}\right)\right] &= \limsup_{n\to\infty}\frac{\lfloor rn\rfloor}{n}\frac{1}{\lfloor rn\rfloor}\log\left[1 + R^{s,\zeta_n}_{\mathrm{SEP}}\left(\rho^{\otimes\lfloor rn\rfloor}\right)\right] \\ &= r\limsup_{n\to\infty}\frac{1}{\lfloor rn\rfloor}\log\left[1 + R^{s,\zeta_n}_{\mathrm{SEP}}\left(\rho^{\otimes\lfloor rn\rfloor}\right)\right] \\ &\overset{\text{(i)}}{\geq} r\limsup_{n\to\infty}\frac{1}{n^{(r)}}\log\left[1 + R^{s,\zeta^{(r)}_n}_{\mathrm{SEP}}\left(\rho^{\otimes n^{(r)}}\right)\right] \\ &\overset{\text{(ii)}}{\geq} r\limsup_{n\to\infty}\frac{1}{n^{(r)}}\log\left[1 + R^{s,\zeta^{(r)}_n}_{\mathrm{SEP}}\left(\rho^{\otimes n}\right)\right] \\ &\overset{\text{(iii)}}{\geq} r\limsup_{n\to\infty}\frac{1}{n+\lceil r\rceil}\log\left[1 + R^{s,\zeta^{(r)}_n}_{\mathrm{SEP}}\left(\rho^{\otimes n}\right)\right] \\ &\overset{\text{(iv)}}{\geq} r\inf_{(\zeta'_n)_n}\left\{\limsup_{n\to\infty}\frac{1}{n}\log\left[1 + R^{s,\zeta'_n}_{\mathrm{SEP}}\left(\rho^{\otimes n}\right)\right] \;\middle|\; \lim_{n\to\infty}\zeta'_n = 0\right\}, \end{aligned} \tag{S97}$$

where: (i) follows since $(n^{(r)})_n$ is simply a subsequence of $(\lfloor rn\rfloor)_n$, up to possible repetitions, and for any l such that $n^{(r)} = \lfloor rl\rfloor$, it holds that $\zeta^{(r)}_n \geq \zeta_l$ by definition; (ii) $n^{(r)} \geq n$ and discarding copies can only decrease R^s_{SEP} (as per Axiom III, clearly obeyed by the resource theory of entanglement); (iii) is a consequence of the fact that

$$n^{(r)} - \lceil r\rceil = \lfloor rl\rfloor - \lceil r\rceil \leq \lfloor rl - r\rfloor = \lfloor r(l-1)\rfloor \leq n \tag{S98}$$

since each $n^{(r)}$ is the *least* value of $\lfloor rl\rfloor$ such that $\lfloor rl\rfloor \geq n$; finally, (iv) follows since adding a constant in the denominator will not affect the asymptotic limit, and $(\zeta^{(r)}_n)_n$ is still a vanishing error sequence. This shows Eq. (S94) and thus concludes the proof of the Lemma. ∎

Proof of Theorem 2. In [7, Theorem S9] we showed that $E^{p=1}_{d,\mathrm{NE}}(\omega_3) = D^{\infty}_{\mathrm{SEP}}(\omega_3) = \log\frac{3}{2}$. Lemma S8 then gives that $E^{p>0}_{d,\mathrm{ANE},N}(\omega_3)$ equals the same value.

On the other hand, in [7, Theorem S9] we also showed that $E^{p=1}_{c,\mathrm{NE}}(\omega_3) = E_\tau(\omega_3) = 1$. By Lemma S9, $E^{p>0}_{c,\mathrm{ANE},N}(\omega_3)$ is the same and in particular is strictly larger than the distillable entanglement. ∎

---

[1] F. G. S. L. Brandão and M. B. Plenio, *A Generalization of Quantum Stein's Lemma*, Commun. Math. Phys. **295**, 791 (2010).

[2] F. G. S. L. Brandão and G. Gour, *Reversible framework for quantum resource theories*, Phys. Rev. Lett. **115**, 070503 (2015).

[3] N. Datta, *Max-relative entropy of entanglement, alias log robustness*, Int. J. Quantum Inform. **07**, 475 (2009).

[4] M. Piani, *Relative Entropy of Entanglement and Restricted Measurements*, Phys. Rev. Lett. **103**, 160504 (2009).

[5] G. Gour, I. Marvian, and R. W. Spekkens, *Measuring the quality of a quantum reference frame: The relative entropy of frameness*, Phys. Rev. A **80**, 012307 (2009).

[6] G. Vidal and R. Tarrach, *Robustness of entanglement*, Phys. Rev. A **59**, 141 (1999).

[7] L. Lami and B. Regula, *No second law of entanglement manipulation after all*, Nat. Phys. **19**, 184 (2023).

[8] F. G. S. L. Brandão and M. B. Plenio, *Entanglement theory and the second law of thermodynamics*, Nat. Phys. **4**, 873 (2008).

[9] F. G. S. L. Brandão and M. B. Plenio, *A Reversible Theory of Entanglement and its Relation to the Second Law*, Commun. Math. Phys. **295**, 829 (2010).

[10] T. Theurer, N. Killoran, D. Egloff, and M. Plenio, *Resource Theory of Superposition*, Phys. Rev. Lett. **119**, 230401 (2017).

[11] J. R. Seddon, *Advancing Classical Simulators by Measuring the Magic of Quantum Computation*, Ph.D. thesis, University College London (2022).

[12] C. Fuchs and J. van de Graaf, *Cryptographic distinguishability measures for quantum-mechanical states*, IEEE Trans. Inf. Theory **45**, 1216 (1999).

[13] A. M. Alhambra, J. Oppenheim, and C. Perry, *Fluctuating States: What is the Probability of a Thermodynamical Transition?* Phys. Rev. X **6**, 041016 (2016).

[14] B. Regula, *Convex geometry of quantum resource quantification*, J. Phys. A: Math. Theor. **51**, 045303 (2018).

[15] M. Tomamichel, *Quantum Information Processing with Finite Resources* (Springer, 2016).

[16] B. Regula, L. Lami, and M. M. Wilde, *Overcoming entropic limitations on asymptotic state transformations through probabilistic protocols*, Phys. Rev. A **107**, 042401 (2023).

[17] M. Horodecki and P. Horodecki, *Reduction criterion of separability and limits for a class of distillation protocols*, Phys. Rev. A **59**, 4206 (1999).

[18] A. W. Harrow and M. A. Nielsen, *Robustness of quantum gates in the presence of noise*, Phys. Rev. A **68**, 012308 (2003).

[19] E. M. Rains, *A semidefinite program for distillable entanglement*, IEEE Trans. Inf. Theory **47**, 2921 (2001).

[20] G. Vidal and R. F. Werner, *Computable measure of entanglement*, Phys. Rev. A **65**, 032314 (2002).
